# Supplementary material for: Dopamine and serotonin in human substantia nigra track social context and value signals during economic exchange
Source: Nat Hum Behav. 2024 Feb 26;8(4):718–28. doi: 10.1038/s41562-024-01831-w (PMC11045309; doi:10.1038/s41562-024-01831-w)
Supplement: Supplementary file 1 — Supplementary Fig. 1 and Table 1. [file 41562_2024_1831_MOESM1_ESM.pdf]

nature human behaviour

Article https://doi.org/10.1038/s41562-024-01831-w

Dopamine and serotonin in human substantia nigra track social context and value signals during economic exchange

In the format provided by the authors and unedited

Table of contents

Page 2. Supplementary Fig. 1. Electrochemical approach.

Page 3. Supplementary Table 1. Patient information.

Page 4. References

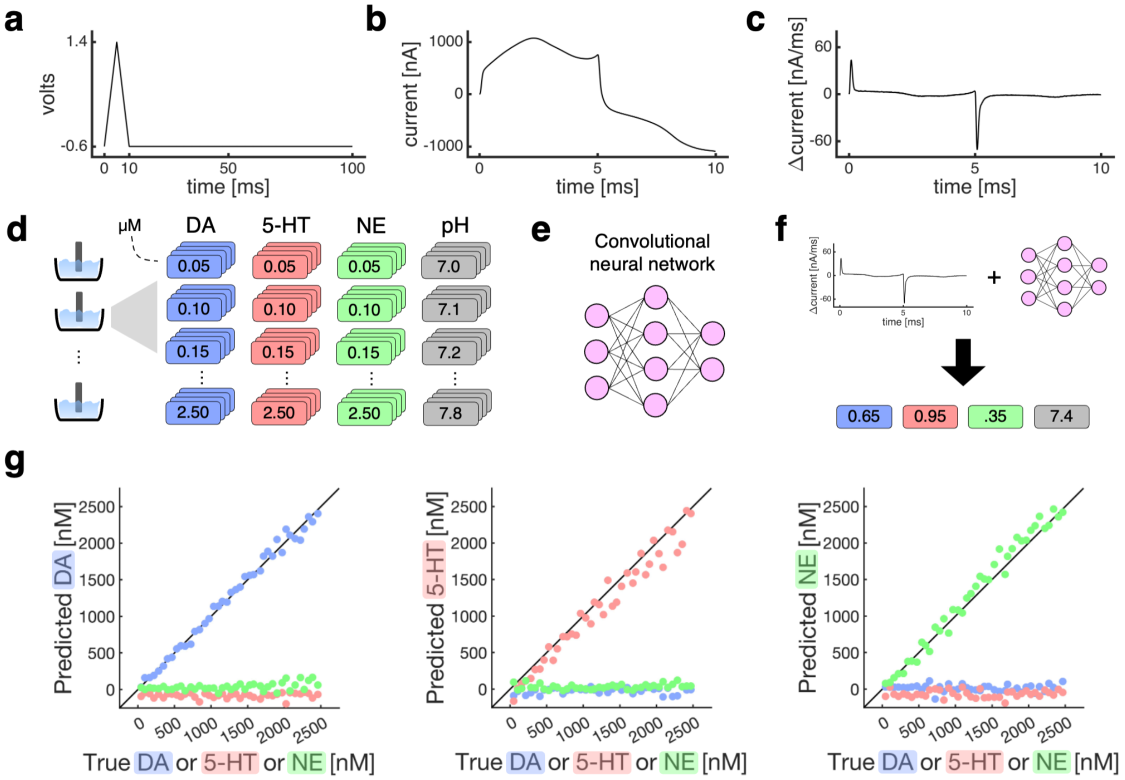

Supplementary Fig. 1. Electrochemical approach. a, The data acquisition protocol is based on fast-scan cyclic voltammetry. We applied a triangular voltage waveform at 10 Hz. **b**, We measured current during the application of the triangular voltage waveform. The current response carries information about the identity and the concentration of analytes in the surrounding neural tissue. **c**, We did not apply background subtraction or decompose the current response into principal components; instead, we used the full (differentiated) current response for signal prediction. **d**, The signal prediction model was trained and tested on in vitro datasets from 64 carbon-fibre electrodes; 59 datasets were used for training and 5 datasets were used for evaluation. For each dataset, dopamine (DA), serotonin (5-HT), norepinephrine (NE) and pH were varied in small increments and multiple measurements were made at each step. In total, the training set consisted of 7,260 unique concentration combinations and 1,089,000 current sweeps, and the test set consisted of 795 unique concentration combinations and 119,250 current sweeps. **e**, The signal prediction model is based on deep convolutional neural networks for time series classification. **f**, The signal prediction model generates concurrent predictions about DA, 5-HT, NE and pH for each current sweep. **g**, In vitro evaluation of the signal prediction model was performed using the datasets withheld from model training. The plots show the predicted concentration (y-axis) of an analyte (colours) as a function of the labelled concentration (x-axis) of each analyte. Black line indicates the “x = y” identity line. Mean data are shown.

Patients			1	2	3	4
Demographic information	Age		80	65	72	69
	Sex		F	M	M	M
	Race		White	White	White	White
	Primary diagnosis		PD/postural instability	PD	PD/tremor dominant	PD/tremor dominant
PD severity	MDS-UPDRS-III	*On*	6 (Mild)	20 (Mild)	20 (Mild)	15 (Mild)
		Off	30 (Mild)	24 (Mild)	55 (Moderate)	32 (Mild)
Psychiatric symptoms	BDI-II		10 (Normal)	10 (Normal)	3 (Normal)	7 (Normal)
	BAI		23 (Moderate)	10 (Normal)	10 (Normal)	10 (Normal)
	Starkstein Apathy Scale		7 (Normal)	9 (Normal)	3 (Normal)	10 (Normal)
Cognitive ability	WASI-II Similarities		24 (Average)	24 (Low average)	21 (Low average)	34 (High average)
	TOPF		65 (Superior)	59 (High average)	*Test not administered*	58 (High average)
Medication history	Carbidopa (mg)		325	112.5	300	350
	Levodopa (mg)		1300	450	1200	1400
	Other medications		Lorazepam<br>Sertraline<br>Gabapentin<br>Mirtazapine<br>Docusate sodium	Amantadine<br>Quetiapine<br>Losartan<br>Pravastatin	Pramipexole<br>Entecavir<br>Rosuvastatin<br>Tacrolimus	Entacapone<br>Amantadine<br>Mirtazapine<br>Fludrocortisone<br>Infliximab<br>Baclofen

Supplementary Table 1. Patient information. Patients had mild to moderate Parkinson's disease (PD) as measured using MDS-UPDRS-III[1]. Patients did not meet diagnostic thresholds for depression, anxiety, or apathy as measured using BDI-II[2], BAI[3], and the Starkstein Apathy Scale[4], respectively. Patients had a range of cognitive scores from low average to superior as measured using WASI-II Similarities[5] and TOPF[6]. All patients received dopamine replacement therapy (carbidopa and levodopa) but different Parkinson and psychiatric medications; all patients were off dopamine replacement therapy and Parkinson medications during the surgical sessions.

References

1. Goetz, C. G. *et al.* Movement Disorder Society-sponsored revision of the Unified Parkinson's Disease Rating Scale (MDS-UPDRS): scale presentation and clinimetric testing results. *Mov. Disord.* **23**, 2129–2170 (2008).
2. Beck, A.T., Steer, R.A., & Brown, G. *Beck Depression Inventory-II*. (Psychological Corporation, 1996).
3. Aaron T. Beck & Robert A. Steer. *BAI: Beck Anxiety Inventory: Manual.* (Psychological Corporation, 1996).
4. Starkstein, S. E. *et al.* Reliability, validity, and clinical correlates of apathy in Parkinson's disease. *The Journal of Neuropsychiatry and Clinical Neurosciences* **4**, 134–139 (1992).
5. Wechsler, D. *Wechsler Abbreviated Scale of Intelligence: WASI-II: Manual*. (Pearson, 2011).
6. Holdnack, J.A. & Drozdick, L.W. *Advanced Clinical Solutions for Use with WAIS-IV and WMS–IV*. (Pearson, 2009).
