# Supplementary material for: Dopamine and serotonin in human substantia nigra track social context and value signals during economic exchange
Source: Nat Hum Behav. 2024 Feb 26;8(4):718–28. doi: 10.1038/s41562-024-01831-w (PMC11045309; doi:10.1038/s41562-024-01831-w)
Supplement: Supplementary file 2 — Reporting Summary [file 41562_2024_1831_MOESM2_ESM.pdf]

nature portfolio

Corresponding author(s): Dan Bang, Seth R. Batten, Xiaosi Gu, P. Read Montague

Last updated by author(s): Jan 10, 2024

Reporting Summary

Nature Portfolio wishes to improve the reproducibility of the work that we publish. This form provides structure for consistency and transparency in reporting. For further information on Nature Portfolio policies, see our Editorial Policies and the Editorial Policy Checklist.

Statistics

For all statistical analyses, confirm that the following items are present in the figure legend, table legend, main text, or Methods section.

n/a	Confirmed	
☐	☒	The exact sample size (*n*) for each experimental group/condition, given as a discrete number and unit of measurement
☐	☒	A statement on whether measurements were taken from distinct samples or whether the same sample was measured repeatedly
☐	☒	The statistical test(s) used AND whether they are one- or two-sided<br>*Only common tests should be described solely by name; describe more complex techniques in the Methods section.*
☐	☒	A description of all covariates tested
☒	☐	A description of any assumptions or corrections, such as tests of normality and adjustment for multiple comparisons
☐	☒	A full description of the statistical parameters including central tendency (e.g. means) or other basic estimates (e.g. regression coefficient) AND variation (e.g. standard deviation) or associated estimates of uncertainty (e.g. confidence intervals)
☐	☒	For null hypothesis testing, the test statistic (e.g. *F*, *t*, *r*) with confidence intervals, effect sizes, degrees of freedom and *P* value noted<br>*Give P values as exact values whenever suitable.*
☒	☐	For Bayesian analysis, information on the choice of priors and Markov chain Monte Carlo settings
☐	☒	For hierarchical and complex designs, identification of the appropriate level for tests and full reporting of outcomes
☐	☒	Estimates of effect sizes (e.g. Cohen's *d*, Pearson's *r*), indicating how they were calculated

Our web collection on statistics for biologists contains articles on many of the points above.

Software and code

Policy information about availability of computer code

Data collection	The experimental task was programmed in PsychoPy 2021.1.4.<br><br>The behavioral data were recorded using PsychoPy 2021.1.4.<br><br>The neural data were recorded using pCLAMP 10 Axon Instruments.
Data analysis	Dopamine and serotonin signal predictions were generated using a custom implementation of the InceptionTime time series classification model (Fawaz et al., Data Mining and Knowledge Discovery, 2020) in Python 3.9.7 using TensorFlow 2.6.0 and Keras 2.6.0.<br><br>Behavioral and neural data were analyzed using standard statistical tests as implemented in MATLAB R2015b and MATLAB R2023a.<br><br>Code for reproducing figres is available on GitHub: https://github.com/danbang/article-DA-5HT-UG-SNr.

For manuscripts utilizing custom algorithms or software that are central to the research but not yet described in published literature, software must be made available to editors and reviewers. We strongly encourage code deposition in a community repository (e.g. GitHub). See the Nature Portfolio guidelines for submitting code & software for further information.

Data

Policy information about availability of data

All manuscripts must include a data availability statement. This statement should provide the following information, where applicable:

- Accession codes, unique identifiers, or web links for publicly available datasets
- A description of any restrictions on data availability
- For clinical datasets or third party data, please ensure that the statement adheres to our policy

Behavioral and neural data are available on GitHub: https://github.com/danbang/article-DA-5HT-UG-SNr.

Research involving human participants, their data, or biological material

Policy information about studies with human participants or human data. See also policy information about sex, gender (identity/presentation), and sexual orientation and race, ethnicity and racism.

Reporting on sex and gender	We report the sex, age and disease of each participant. All participants have the same disease (Parkinson's disease). Sex and age were not included as covariates in any analysis. Details are provided in Supplementary Table 1.
Reporting on race, ethnicity, or other socially relevant groupings	We report the sex, age and disease status of each participant. All participants have the same disease (Parkinson's disease). Sex and age were not included as covariates in any analysis. Details are provided in Supplementary Table 1.
Population characteristics	Participants (n = 4, 1 female, mean age +/- SE = 71.3 +/- 3.4 years) were Parkinson's disease patients who underwent awake brain surgeries for the bilateral implantation of DBS electrodes in the subthalamic nucleus of each hemisphere.
Recruitment	Participants were recruited among Parkinson's disease patients who were scheduled to undergo awake brain surgeries for the bilateral implantation of DBS electrodes in the subthalamic nucleus of each hemisphere. Once they had agreed to the clinical treatment, they were assessed for suitability for the research study and given the option to participate. Before obtaining informed written consent, the research team provided both written and verbal information about the research study and how it would alter the clinical procedure. Specifically, patients were informed that the study would involve a research-exclusive probe (carbon-fiber electrode) and that extra time (maximum 30 min) would be needed to complete the study. This information was provided both verbally and in a written document. Patients did not receive compensation for participation, and they knew that they would not receive any money earned in the task. The recruitment procedure may have selected for patients who are very keen to contribute to science.
Ethics oversight	The study was approved by the IRB committees at the Icahn School of Medicine at Mount Sinai (13-00415) and Virginia Tech (11-078).

Note that full information on the approval of the study protocol must also be provided in the manuscript.

Field-specific reporting

Please select the one below that is the best fit for your research. If you are not sure, read the appropriate sections before making your selection.

☒ Life sciences ☐ Behavioural & social sciences ☐ Ecological, evolutionary & environmental sciences

For a reference copy of the document with all sections, see nature.com/documents/nr-reporting-summary-flat.pdf

Life sciences study design

All studies must disclose on these points even when the disclosure is negative.

Sample size	The opportunity to perform human electrochemistry as part of DBS surgery is rare. We collected the maximum number of participants possible within the study period. The sample size is in line with previous studies performing human electrochemistry as part of DBS surgery (e.g., Bang et al., Neuron, 2020).
Data exclusions	We excluded trials where the choice reaction time was longer than 14 s to minimize the impact of any momentary distraction in the operating room (around 5% of trials). In analyses of history effects, we excluded trials preceded by an already excluded trial and the first trial of a block.
Replication	The experimental findings were evaluated using statistical testing and associated test statistics and not through replication.
Randomization	Each participant performed the ultimatum game twice in two separate surgical sessions. For each participant, the human versus computer conditions were blocked within a session (2 conditions x 30 trials = 60 trials in a session) and counterbalanced across sessions. The order of the human versus computer conditions in the first session was counterbalanced across participants.
Blinding	Blinding was not relevant for the current study as awareness of the experimental conditions was a critical part of the study design.

Reporting for specific materials, systems and methods

We require information from authors about some types of materials, experimental systems and methods used in many studies. Here, indicate whether each material, system or method listed is relevant to your study. If you are not sure if a list item applies to your research, read the appropriate section before selecting a response.

Materials & experimental systems

n/a	Involved in the study	
☒	☐	Antibodies
☒	☐	Eukaryotic cell lines
☒	☐	Palaeontology and archaeology
☒	☐	Animals and other organisms
☒	☐	Clinical data
☒	☐	Dual use research of concern
☒	☐	Plants

Methods

n/a	Involved in the study	
☒	☐	ChIP-seq
☒	☐	Flow cytometry
☒	☐	MRI-based neuroimaging
